# Unilateral severe gynecomastia in a 14 year-old adolescent with neurofibromatosis type 1 undergoing endoscopic mastectomy: a case report

**DOI:** 10.3389/fmed.2024.1364089

**Published:** 2024-07-01

**Authors:** Fangjian Shang, Bo Xi, Duojun Qiu, Xin Chen, Yifang Wang, Meng He, Bo Liu, Zengren Zhao

**Affiliations:** The First Hospital of Hebei Medical University, Shijiazhuang, China

**Keywords:** gynecomastia, NF-1, endoscopy, mastectomy, adolescent

## Abstract

Gynecomastia can be caused by neurofibromas but has rarely been reported. The present case report describes the clinical appearance, diagnosis, and therapy of a rare combination of a 14 year-old adolescent male unilateral severe gynecomastia with NF-1 neurofibromatosis. In this particular case, we successfully performed minimally invasive surgery using endoscopic mastectomy, which not only resulted in a satisfactory appearance but also confirmed the presence of neurofibroma type 1 by detecting typical immunohistochemical indicators associated with the disease. Additionally, we analyzed the gene responsible for the disease, c.1431del: p. F477Lfs*21, based on the patient’s family history.

## Introduction

Gynecomastia is a condition characterized by the benign proliferation of male breast tissue, which can occur at various stages of life. The causes of breast development can be categorized as physiological or pathological. Physiological breast development typically occurs during specific life stages in males, including infancy, adolescence, and old age (1). It is believed to be due to an alteration in the androgen-estrogen ratio and the effects of other hormones such as growth hormone, insulin-like growth factor 1, and prolactin, along with factors affecting the aromatase enzyme, on the other hand, pathological breast development may be linked to factors like medication use, certain medical conditions, endocrine disorders, and obesity. Several diseases, including liver or renal failure, thyrotoxicosis, Klinefelter syndrome, tumors, and exposure to environmental pollutants, can also cause gynecomastia (1, 2). Gynecomastia typically presents with either bilateral or unilateral breast enlargement. While unilateral breast development is less common, previous studies have indicated that conditions such as Peutz-Jeghers syndrome can potentially lead to this occurrence (2, 3).

Neurofibromatosis (NF) is a progressive autosomal dominant disease that affects various systems of the body. It is classified into four types, with neurofibromatosis type I being the most common, accounting for approximately 96% of NF patients. Neurofibromatosis type II has an incidence rate of about 3% (4). Additionally, there are two atypical and rare types of neurofibromas, with an incidence rate generally less than 1% (5). The NF1 type exhibits several typical clinical features, including café-au-lait macules (CALMs) plexiform neurofibromas, optic pathway glioma, bone development abnormalities (such as scoliosis), and learning disabilities. Axillary or inguinal freckles are also observed. Among these symptoms, neurofibroma is the most common and characteristic (6).

This article reports a rare case of a 14 year-old patient with concurrent neurofibromatosis and unilateral severe gynecomastia.

## Case report

**Figure 1:**
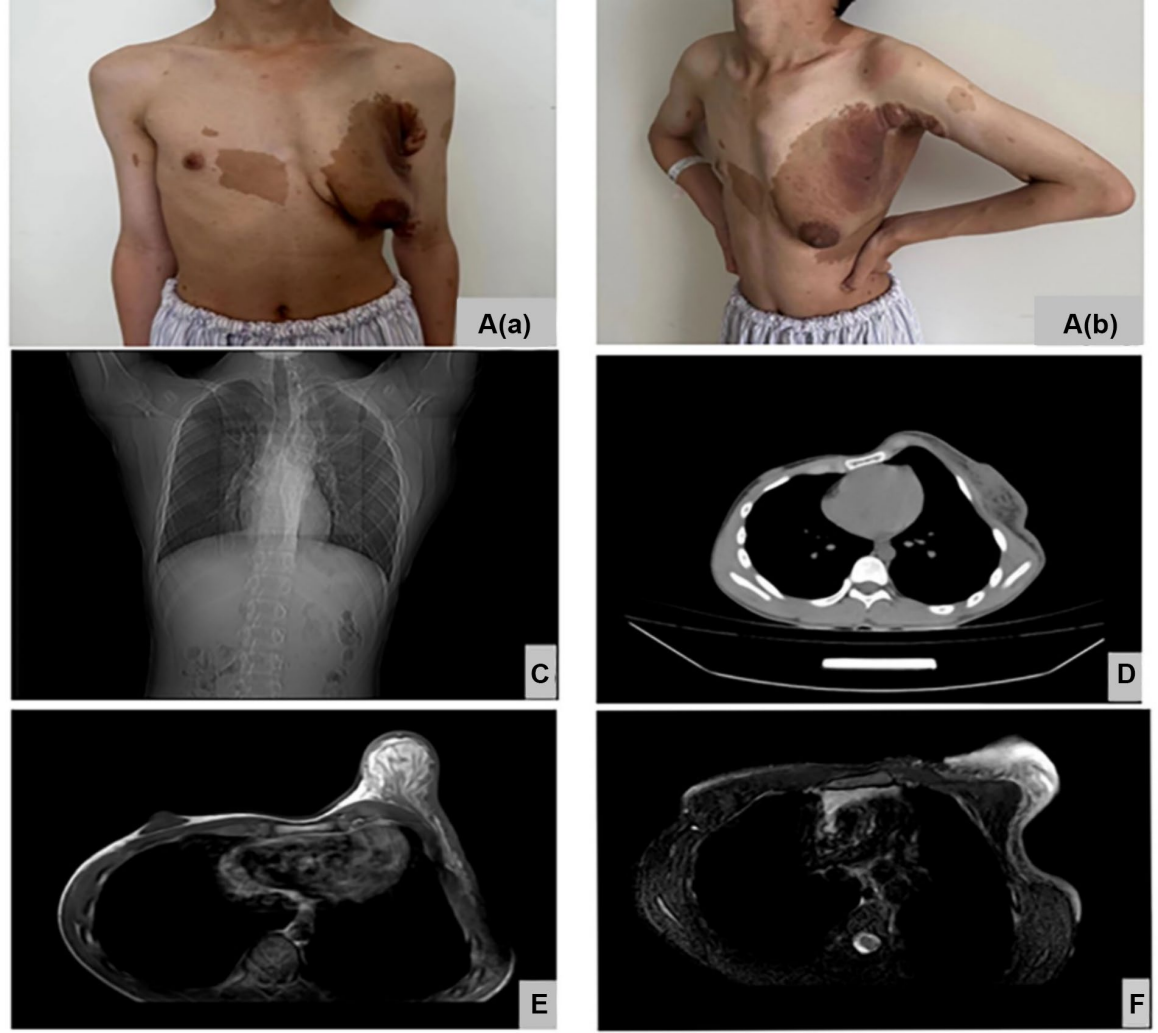
Shown the front view of patient **(A(a))** Lateral view of patient **(A(b))**, CT showing thoracic scoliosis and scoliosis **(B)**, CT showing increased glands in the left breast **(C)**, Breast MRI showing male breast development **(D)**, Breast MRI showing edema in the left breast, skin and anterior chest wall **(E)**.

The patient is a 14 year-old male adolescent who presented with a complaint of progressive enlargement of his left breast over a period of 13 years. Upon physical examination, it was observed that the chest was lordotic and the spine was convex to the left. The left breast appeared enlarged and sagging, with multiple irregular café-au-lait spots on the chest wall (more than 6 spots on the entire body). There were no signs of redness, swelling, or ulceration on the skin. The left nipple-areola complex was significantly enlarged, with a diameter of approximately 4 cm for the areola and 1 cm for the nipple. No depression or discharge was observed from the nipple. The palpable tough tissue in the left breast measured approximately 9.0 cm × 6.0 cm × 5.0 cm (Figures 1Aa,b). The patient presented with gynecomastia as the primary reason for seeking medical attention, having not received any prior treatment. Following admission, a chest CT examination was conducted, revealing thoracic scoliosis (Figure 1B) and increased glandular tissue in the left breast (Figure 1C). MRI results of both breasts indicated the presence of gynecomastia (Figure 1D), with edema observed in the left breast, as well as the skin and front chest wall (Figure 1E). Routine auxiliary blood tests, as well as hormone and inflammatory factor tests, were performed (Table 1).

**Table 1:** Initial laboratory assessment.

Laboratory tests	Result
FSH (mIU/L)	1.95 (1.27–19.26)
LH (mIU/L)	1.78 (1.24–8.62)
PRL (ng/mL)	6.59 (2.64–13.13)
Testo (ng/mL)	6.73 (1.75–7.81)
Prog (ng/mL)	0.25 (0.10–0.84)
E2 (pg/mL)	21.49 (0.00–38.95)
IL-1β (pg/mL)	2.69 (0.00–12.40)
IL-2 (pg/mL)	1.39 (0.00–5.71)
IL-4 (pg/mL)	1.52 (0.00–3.00)
IL-5 (pg/mL)	0.89 (0.00–3.10)
IL-6 (pg/mL)	7.56 (0.00–7.00)
IL-8 (pg/mL)	1,567 (0.00–20.60)
IL-10 (pg/mL)	2.35 (0.00–4.91)
TNF-α (pg/mL)	2.90 (0.00–4.60)
TNF-γ (pg/mL)	1.55 (0.00–7.42)
IL-17A (pg/mL)	1.17 (0.00–20.60)
IL-12P70 (pg/mL)	0.66 (0.00–3.40)
IFN-α (pg/mL)	1.41 (0.00–8.50)

**Figure 2:**
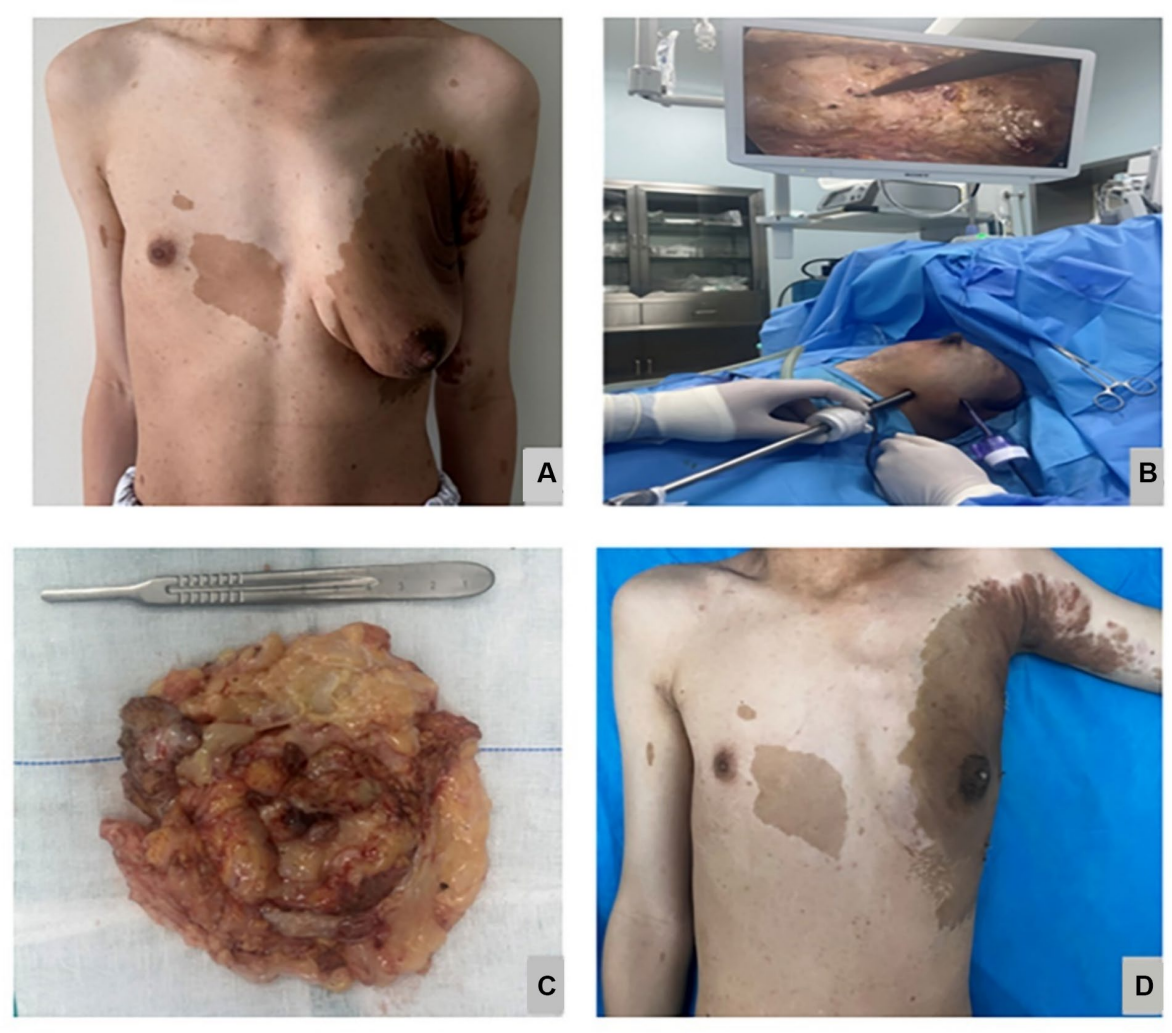
The preoperative appearance is shown in **(A)**, the intraoperative minimally invasive breast endoscopic resection of the tumor is shown in **(B)**, the Gross specimen of the tumor is shown in **(C)**, the postoperative appearance 2 weeks is shown in **(D)**.

**Figure 3:**
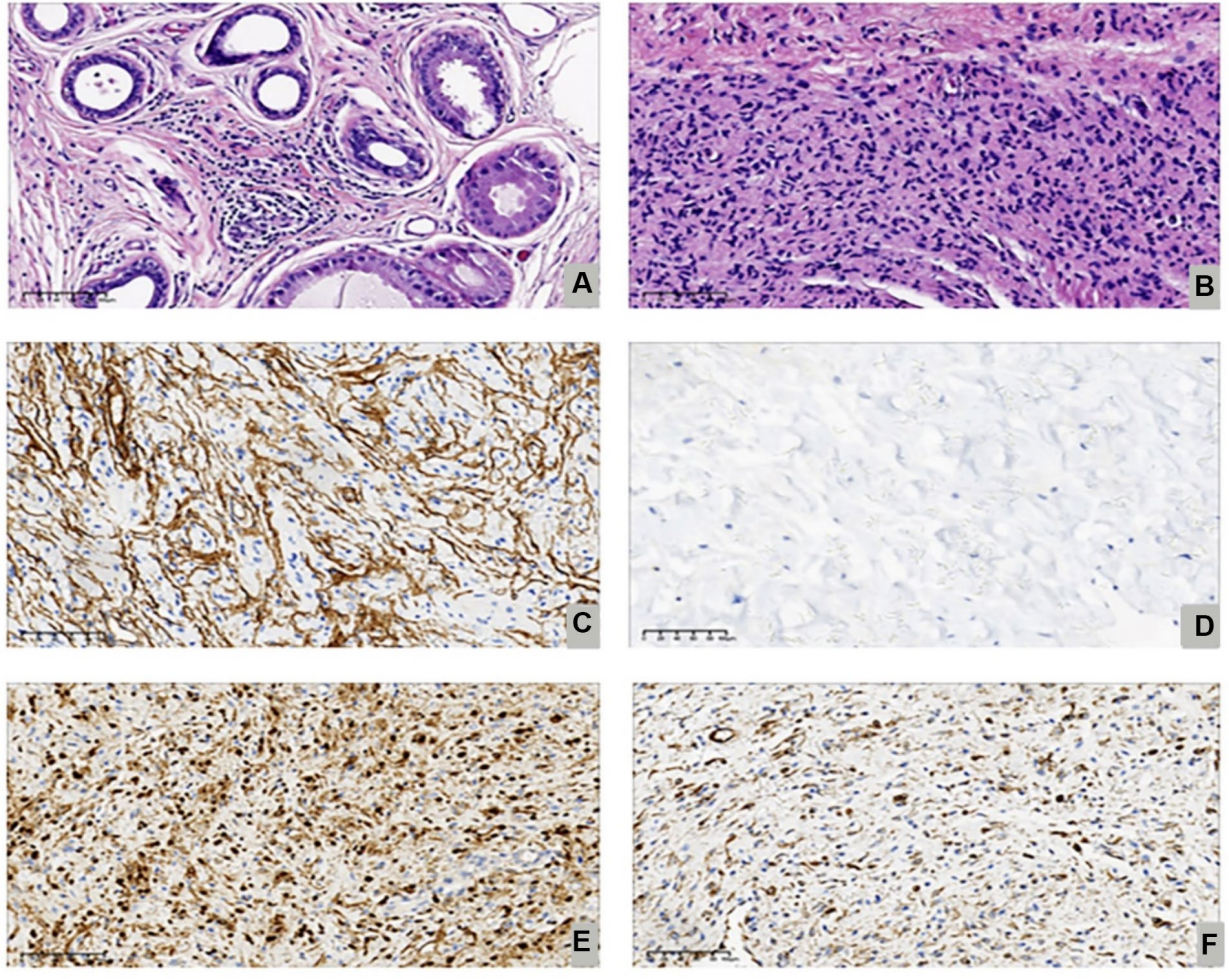
Shown the proliferative phase with ductal epithelial proliferation, periductal edema and vascularized stroma (Hematoxylin Eosin, 200X) **(A)**, focal spindle cell areas reminiscent of neurofibroma (Hematoxylin Eosin, 200X) **(B)**, Immunostaining for CD34 (200X) **(C)**, Immunostaining for NF-1(200X) **(D)**, Immunostaining forS-100(200X) **(E)**. Immunostaining for Vim (200X) **(F)**.

After consultation with the patient, we opted to perform minimally invasive breast endoscopy surgery to remove the local tissue using a combination of endoscopy mastectomy and liposuction (Liu and Shang’s 2 hole 7-step method) (Figure 2) (7). The postoperative pathology report indicated the presence of gynecomastia and neurofibroma (Figures 3A,B). Immunohistochemistry results (Table 2) showed S-100(+), NF(−), Vim(+), CD34(+), which led to the patient being diagnosed with NF-1 neurofibromatosis (Figures 3C–F).

**Table 2:** Summary of immunohistochemistry.

Antibodies to	Result
S-100	Positive
NF	Negative
EMA	Positive
CK	Negative
Vim	Positive
S-100	Positive
NF	Negative
SOX-10	Positive
Ki-67	Positive
EMA	Positive
ERG	Positive
CD34	Positive
CD68	Negative
SMA	Negative
Des	Negative
P16	Positive
P53	Negative
HMB45	Negative
Melan-A	Positive

**Figure 4:**
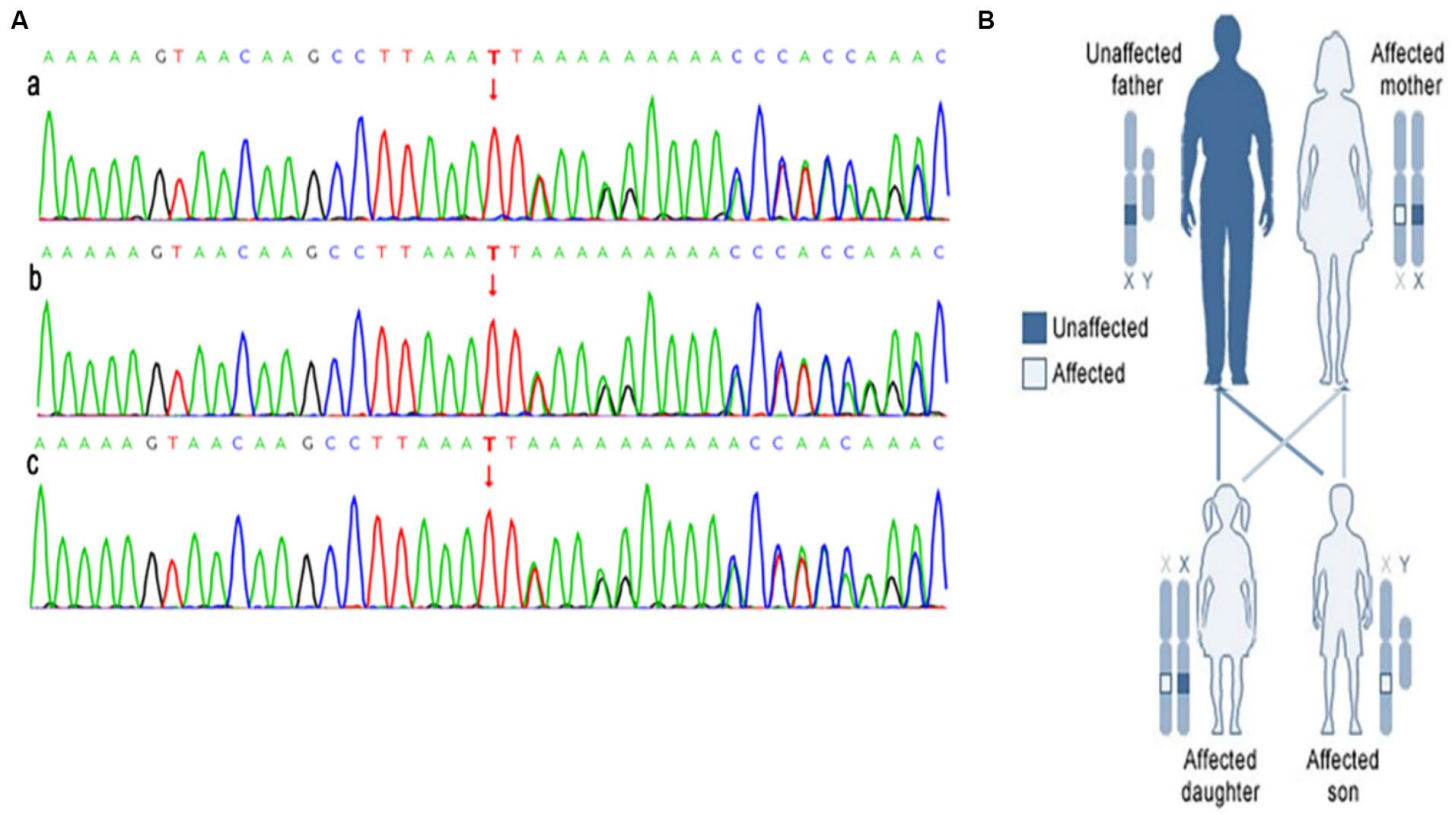
The results of the next generation sequencing of the NF1 gene is shown in **(A)**, **a** is gene sequence of patient (heterozygosis); **b** is the patient’s mother’s gene sequence (heterozygosis); **c** is patient’s sister’s gene sequence (heterozygosis). The NF1 affects members in each generation of a family is shown in **(B)**.

Further investigation into the patient’s family history revealed that his father was in good health, while his mother and sister exhibited multiple flaky café-au-lait spots all over their skin and were both diagnosed with NF-1 neurofibromatosis. Genetic analysis of the family identified a heterozygous frameshift deletion variant c.1431del:p.F477Lfs*21 in the NF1 gene in all three individuals. This mutation involves the deletion of base 1,431 in the cDNA, resulting in a change from phenylalanine to leucine at codon position 477 and a frameshift leading to a premature stop codon (Figure 4).

## Discussion

Gynecomastia is a prevalent condition that affects boys and men. It is typically characterized by an increase in benign tissue. However, it is important to note that when there is unilateral breast development, it could indicate the presence of malignant lesions, which warrants our attention.

Neurofibromatosis type I and prepubertal gynecomastia have been the subject of numerous case reports. In their study, Murat et al. (8) presented the case of a 10 year-old boy who underwent pathologic examination of a solid breast mass measuring 3 cm, which was confirmed to be neurofibroma type I. Similar findings were reported by Damiani and Eusebi (9), they documented a case of bilateral gynecomastia and neurofibromatosis of the breast in a 6 year-old boy. In another article, six cases of men with gynecomastia and neurofibromatosis were reported, and it is noteworthy that the hormone levels of the six patients mentioned in this article were found to be normal (10). Furthermore, Zamecnik et al. (11) discussed a patient with a surgical history of gynecomastia in the left breast, which was successfully removed. What these cases all have in common is that neurofibromas are a major contributor to gynecomastia. These cases all show that neurofibromas can cause gynecomastia.

The most common cause of NF1 is a mutation in the NF1 gene, which is located on chromosome 17q11.2. The NF1 gene is a large gene spanning 350 kb and consists of 60 exons. It encodes a protein called neurofibromin, composed of 2,818 amino acids, which has tumor suppressor effects (12). A diagnosis of NF1 requires the presence of two or more of the following (5): the presence of six or more cafe´-au-lait spots, two or more neurofibromas or one or more plexiform neurofibromas, freckling in the axilla or groins, optic glioma, two or more Lisch nodules, sphenoid wing dysplasia or thinning of a long bone cortex with or without pseudoarthrosis, and a first-degree relative who also meets the above criteria for NF1. The patient in question fulfilled the diagnostic criteria for NF-1, exhibiting characteristic cafe-au-lait spots and unilateral severe neurofibroma-related gynecomastia. Genetic testing indicated that the patient’s mother and sister also met the diagnostic criteria for NF-1.

Gynecomastia can have a significant impact on the psychological and emotional well-being of patients. In this specific instance, we employed laparoscopic breast surgery techniques to address the condition. Following the procedure, the patient experienced a flattening of the chest and reported a reduction in psychological stress.

We present a detailed case report of a rare patient with NF1 neurofibroma and unilateral severe gynecomastia. The patient underwent laparoscopic breast surgery to successfully remove the tumor, leading to a satisfactory local appearance. Pathology and immunohistochemistry analyses were conducted to confirm the diagnoses of both diseases. Additionally, genetic testing was carried out on the patient’s family members to determine the presence of neurofibromatosis, a dominantly inherited disease.

## Data availability statement

The original contributions presented in the study are included in the article/supplementary material, further inquiries can be directed to the corresponding authors.

## Ethics statement

Written informed consent was obtained from the individual(s), and minor(s)’ legal guardian/next of kin, for the publication of any potentially identifiable images or data included in this article.

## Author contributions

FS: Methodology, Investigation, Conceptualization, Writing – original draft. BX: Writing – original draft, Data curation. DQ: Writing – review & editing. XC: Writing – review & editing. YW: Writing – review & editing. MH: Data curation, Writing – review & editing. BL: Writing – review & editing, Funding acquisition. ZZ: Writing – review & editing, Funding acquisition.
